# Supplementary material for: Study on the Function of SlWRKY80 in Tomato Defense against Meloidogyne incognita
Source: Int J Mol Sci. 2024 Aug 15;25(16):8892. doi: 10.3390/ijms25168892 (PMC11354995; doi:10.3390/ijms25168892)
Supplement: Supplementary file 1 [file ijms-25-08892-s001.zip › ijms-3111864-supplementary.pdf]

International Journal of *Molecular Sciences*

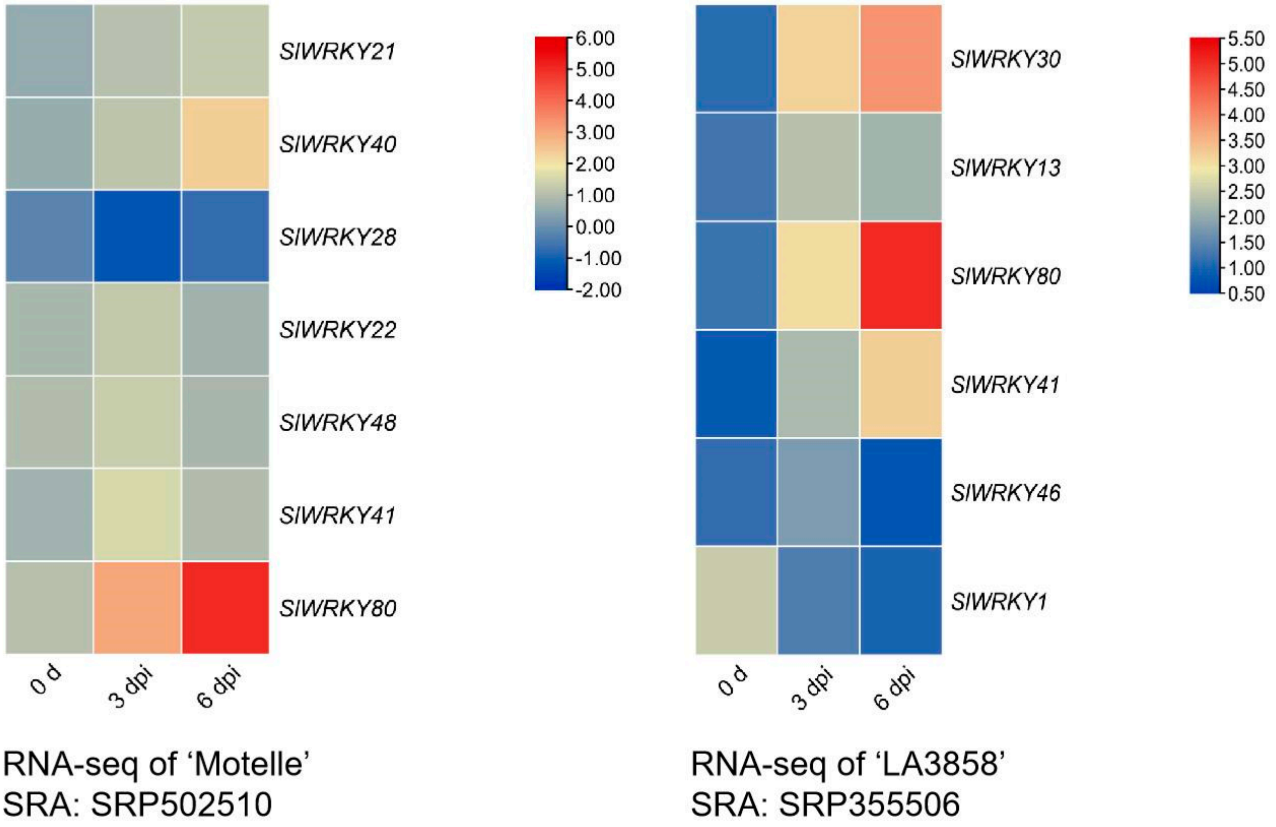

Figure S1. The expression pattern of DEG-encoded WRKYs in the RNA-seq results of 'Motelle' and 'LA3858'.

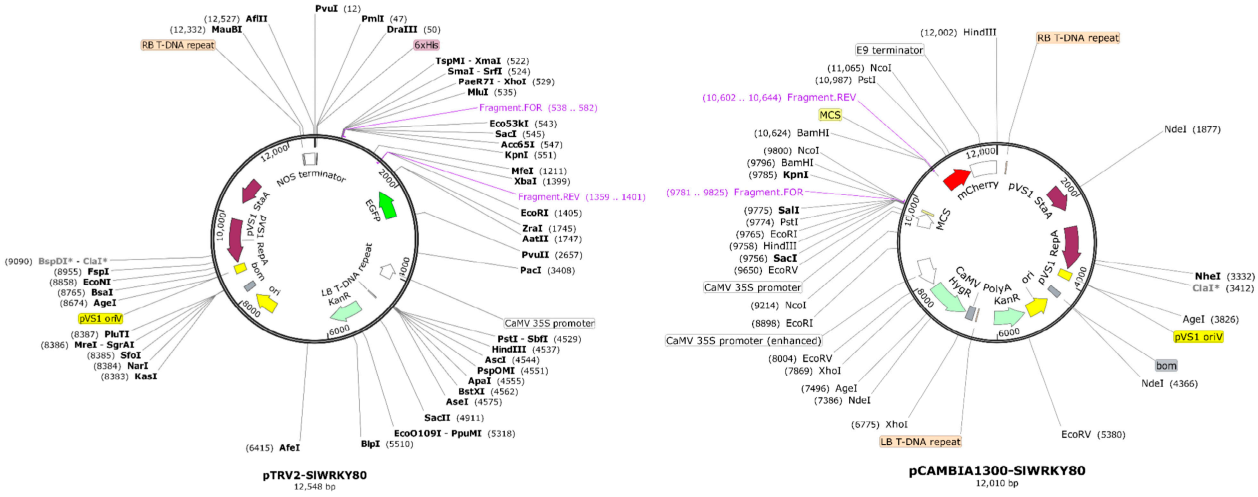

Figure S2. Plasmid map of *SlWRKY80* recombinant with *pTRV2* and *pCAMBIA1300*.

Table S1. Detailed information on the CDS sequence and homologous recombination clone primers of *SlWRKY80*, as well as the primers of the reference gene (*SlActin*) and *SlWRKY80* by RT-qPCR.
